# A Necessary Part of Preventive Medicine

**Published:** 1921-07-16

**Authors:** 


### REACTION AGAINST FEAR.

### A Necessary Part of Preventive Medicine.

The tendency in all widespread movements of reform is to drift into taking the line of least resistance, and it is therefore not surprising that the more hopeful outlook on public health matters which is known as preventive work has begun to show symptoms of degenerating into the cure of light cases. From the moment when disease or a tendency to disease begins to appear the work of prevention may be considered to have definitely failed. To effect a cure before disease gains a firm grip is an exceedingly important part of health work, but it is not prevention.

There is a stage in the history of every patient when he is, to all intents and purposes, in normal health, afflicted by no bad symptoms, indistinguishable from his fellows. In the prevention of disease this is the period it is necessary to study. A hundred workmen are engaged in the same occupation, spending their working hours in the same way, to all appearance not greatly differentiated from each other in their homes. All, without exception, in every occupation are exposed to influences and conditions which are more or less disadvantageous to health. Such influences and conditions are resisted successfully by the majority. A certain percentage, however, succumb. The point immediately preceding this crisis is that with which preventive medicine is concerned. If we would prevent disease we must ascertain more about the factors which break down resistance to it.

Patients are not very good judges of the causes which have weakened their resistance. Their favourite explanation of such diseases as heart or kidney complaints, tuberculosis or cancer, is heredity. The annals of the family history are ransacked, and while it is often considered unnecessary to search further than the consumptive mother or the uncle who died a sudden death, on occasions an astonishing catalogue of disastrous illnesses and early deaths among near relations will be poured out. Before concluding that the existence of disease or a tendency to disease is due to its transmissible qualities, however, it would be necessary to collect well-authenticated instances of heredity in which the victims were unaware of their family history. It is barely possible that useful statistics in this direction might be secured in orphanages, but so morbid is the general outlook that it is to be suspected that, were there but one friend of the hapless orphan acquainted with the alleged cause of his parents' death that sinister surmise would be imparted and enlarged upon. In the matter of heredity it is only too common for children to be brought up under the shadow of a great fear. The ordinary child full of absorbing occupations and healthily disregardful of grown-up talk escapes the morbid influence. The sensitive, receptive child, often the best of the family, grows up "delicate," accustomed to hear this fact always mentioned, and ready to flag on any occasion demanding the calling up of reserve strength. It is these who, when they are exposed later to adverse conditions, are discovered to lack resistance.

It is by no means to be taken for granted that nothing can be done to eliminate the fear of disease from those whom it is sucking down into the whirlpool. The past fifty years, during which so much attention has been centred less on health than on disease, have much to answer for. The very real complaint which is familiarly known as "hospitalism"—the tendency among the inhabitants of a district surrounding a famous hospital to develop symptoms of disease—points to the fact that much brooding upon disease, much conversation about symptoms, much morbid contemplation of diseased processes in others, when not counterbalanced by knowledge and the consciousness of ministering relief to the sufferers tends in itself to arouse the fear which weakens resistance.

Has not the time arrived when the evil effects of fear in weakening the fibres of the mind, and suggesting a deterioration which tends to become habitual, should be taken into consideration and seriously combated? The immediate effect of a well-managed sanatorium under exhilarating and purposeful direction is to banish the horrible dread which darkens the subconscious mind of the consumptive. His whole outlook changes under the hopeful régime which prescribes little but Nature's remedies. Alas! on his return even to the healthiest surroundings, the original atmosphere of suspicion and dread is liable to meet him on the threshold of his home. Can nothing be done to counter this depressing influence?

The need is for more knowledge, more reliance on the latent powers, a saner education. In such centres as the Almoner's department at St. Thomas's the work of educating the people to believe in health and in the forces within them which resist the onset of disease are being systematically taught. Thousands of such centres, thousands of trained workers are needed to counteract the terrible fears which concentration on disease have roused among the ignorant and the educated alike. No house can be set in order without raising a cloud of dust to begin with. In the effort to set in order the nation's health we are suffering badly to-day from the great cloud of fear which has been stirred up about our heads.

